# Splice variant transcripts of the anterior gradient 2 gene as a marker of prostate cancer

**DOI:** 10.18632/oncotarget.2365

**Published:** 2014-08-19

**Authors:** Antje Neeb, Simon Hefele, Stefanie Bormann, Walther Parson, Fabian Adams, Philipp Wolf, Arkadiusz Miernik, Martin Schoenthaler, Malte Kroenig, Konrad Wilhelm, Wolfgang Schultze-Seemann, Sigrun Nestel, Georg Schaefer, Huajie Bu, Helmut Klocker, Irina Nazarenko, Andrew C. B. Cato

**Affiliations:** ^1^ Karlsruhe Institute of Technology, Institute of Toxicology and Genetics, Eggenstein-Leopoldshafen, Germany; ^2^ Department of Environmental Health Sciences and Hospital Infection Control, Medical Center-University of Freiburg, Freiburg, Germany; ^3^ Institute of Legal Medicine, Innsbruck Medical University, Innsbruck, Austria; ^4^ Penn State Eberly College of Science, University Park, PA, USA; ^5^ Department of Urology, Medical Center-University of Freiburg, Freiburg, Germany; ^6^ Institute of Anatomy and Cell Biology, University of Freiburg, Freiburg, Germany; ^7^ Department of Urology, Division of Experimental Urology, Innsbruck Medical University, Innsbruck, Austria; ^8^ Heinrich Heine University, Institute of Toxicology, Düsseldorf, Germany

**Keywords:** Anterior gradient 2 gene, Exosomes, Prostate cancer diagnosis, Urinary biomarkers, Splice variants

## Abstract

Anterior gradient 2 (AGR2) is a gene predominantly expressed in mucus-secreting tissues or in endocrine cells. Its expression is drastically increased in tumors including prostate cancer. Here we investigated whether AGR2 transcript levels can be used as a biomarker to detect prostate cancer (PCa). Using a PCR-based approach, we could show that in addition to the wild-type (AGRwt long and short) transcripts, five other AGR2 splice variants (SV) (referred to as AGR2 SV-C, -E, -F, -G and -H) were present in cancer cell lines. In tissue biopsies, SV-H and AGR2wt (short) distinguished between benign and PCa (p ≤ 0.05 n = 32). In urine exosomes, AGR2 SV-G and SV-H outperformed serum PSA. Receiver operating characteristic (ROC) curves showed the highest discriminatory power of SV-G and SV-H in predicting PCa. AGR2 SV-G and SV-H are potential diagnostic biomarkers for the non-invasive detection of PCa using urine exosomes.

## INTRODUCTION

Prostate cancer (PCa) has become a major public health problem. In many developed countries it is not only the most commonly diagnosed malignancy but also the second leading cause of cancer related deaths in males [1].

Prostate specific antigen (PSA) has been the most valuable tool in the detection, staging and monitoring of prostate cancer [2,3]. Although widely accepted as a prostate tumor marker, it is prostate tissue but not prostate cancer specific as PSA levels have been reported to increase in men with benign prostatic hyperplasia (BPH) and prostatitis [4]. There is therefore an urgent need for molecular tests that can accurately identify men who have early stage, clinically localized prostate cancer. This requires studying the expression patterns of genes in malignant as well as non-malignant prostate tissues; an exercise that has led to the identification of anterior gradient 2 (AGR2) gene [5].

AGR2 was first described in *Xenopus laevis* embryos where it induces the formation of the forebrain and the cement gland [6]. In humans, it is predominantly expressed in mucus-secreting tissues or in endocrine cells where it is implicated in protein folding as a member of protein disulfide isomerase family of endoplasmic reticulum-resident proteins [7]. AGR2 was found to be expressed in estrogen receptor (ER)-positive breast cancer cell lines [8] and at elevated levels in subsets of breast [9], prostate [10–12], non-small cell lung [13], pancreatic [14], and hepatic adenocarcinomas [15].

We have recently demonstrated in immunohistochemical analysis an increased expression of AGR2 in PIN lesions and in low-grade prostate cancers compared to benign tissue [11]. Furthermore we have employed a quantitative real-time polymerase chain reaction (qRT-PCR) method to show the relevance of AGR2 transcript levels in urine sediments as a PCa marker [11]. We have now improved these studies to cover AGR2 transcript levels in urine extracellular membrane vesicles (exosomes) as these samples are usually enriched in tumor-specific transcripts [16]. Additionally, we have included splice transcripts of AGR2 in our study as they are more abundant in tumor tissue for providing growth and survival advantages [17]. These improvements together have led to the identification of two distinct splice variants of AGR2 in urine exosomes as highly significant in distinguishing between benign and prostate cancer. These transcript levels outperformed serum PSA as a marker for prostate cancer in a pilot study on prostate cancer patients.

## RESULTS

**Figure 1:**
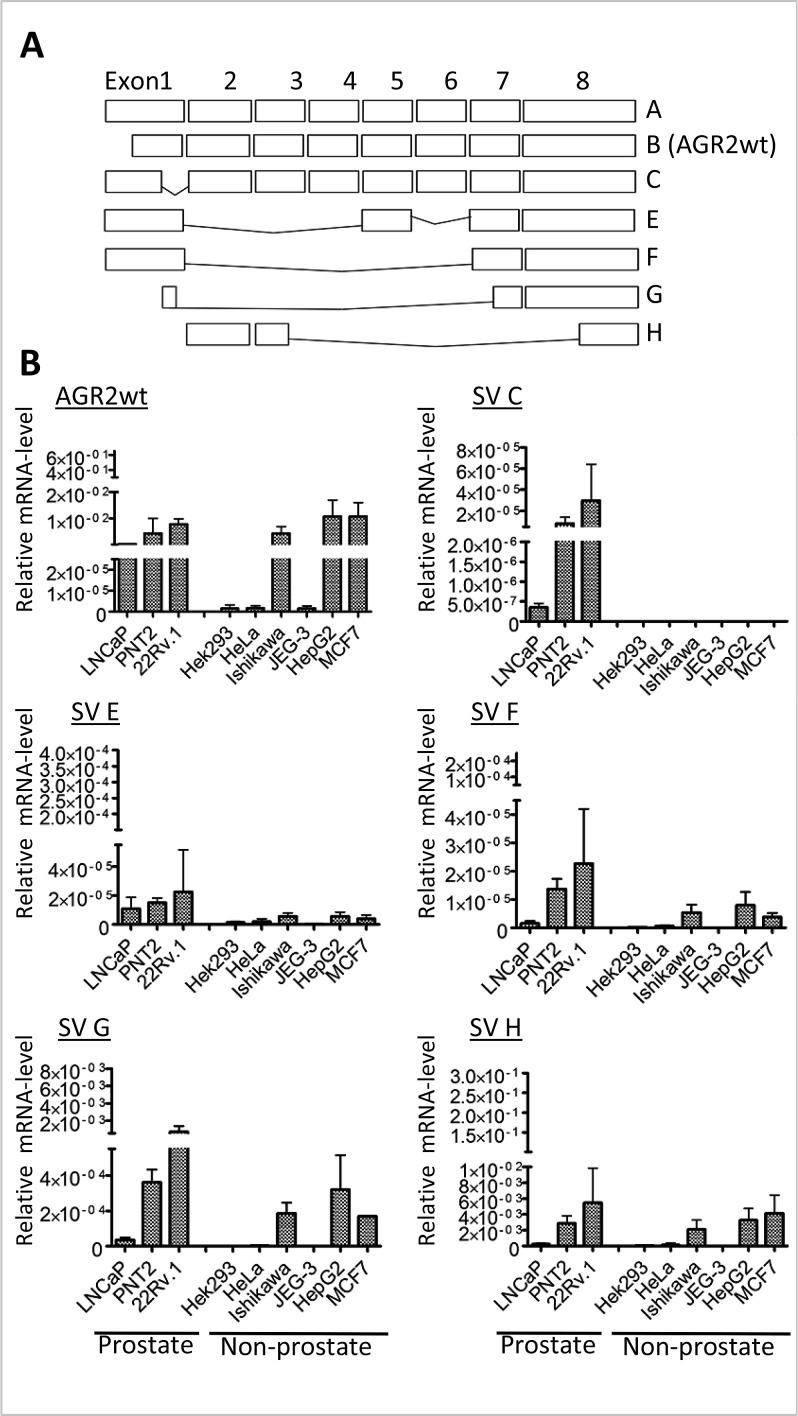
Cell-type specific expression of AGR2 variants (A) Schematic representation of AGR2 wild-type and splice variants cloned from different prostate cancer cells. Exons are presented as rectangles. (B) qRT-PCR showing the level of expression of the different transcripts in prostate and non-prostate cell lines. The expression of the transcripts was determined using splice variant-specific primers and the results are presented relative to the expression of the human ribosomal subunit 36B4. The bar charts are averages of 3 independent experiments ± standard deviation.

AGR2 has a fairly broad expression pattern in human tissues but splice variants of AGR2 (e.g. the long isoform as well as Δ 6 and Δ 4-6 isoforms) are predominantly expressed in certain tissues or in distinct neoplastic situations [15,18]. To determine whether spliced variants exist that are specifically expressed in prostate cancers, we carried out reverse transcribed polymerase chain reaction (rt-PCR) on mRNA isolated from prostate tumor cell lines using primers in exons 1, 2 and 8 of the AGR2 gene. In addition to transcripts A and B that have already been described as the long and short transcripts of the AGR2 gene [18], we cloned and sequenced five new splice transcripts which we termed AGR2 SV-C, AGR2 SV-E, AGR2 SV-F, AGR2 SV-G and AGR2 SV-H (Figure 1A). The newly identified splice variant transcripts most likely arose from alternative splicing as they lack complete exons and do not seem to encode proteins.

**Figure 2:**
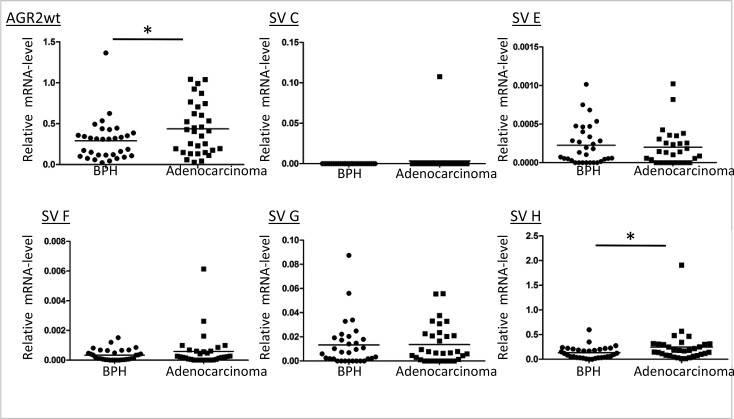
Expression level of AGR2 splice variants in benign and prostate cancer Total RNA isolated from benign and prostate carcinoma biopsies was used for cDNA synthesis and qRT-PCR was carried out with AGR2 splice variant specific primers. The results are presented as scatter plot of the expression level of the AGR2 splice variants relative to the level of expression of the human ribosomal subunit 36B4 (* represents p ≤ 0.05).

We then analyzed the expression of the isolated AGR2 variants in different cell lines to determine their cell type specificity. AGR2wt (AGR2 wildtype short form transcript; AGR2 SV-B) [18] was expressed in nearly all the cell lines we analyzed (Figure 1B). In contrast, AGR2 long isoform (AGR2 SV-A) (Figure 1A) was hardly detected and was therefore excluded from further analysis. The splice variants AGR2 SV-C, -E, -F, -G and -H showed varying levels of expression among the different cell lines while AGR2 SV-C was mainly expressed at low levels only in prostate tumor cell lines (Figure 1B). Quantitative real-time PCR (qRT-PCR) analysis of the AGR2 transcripts was further performed on RNA isolated from radical prostatectomy biopsies. Here only AGR2wt and AGR2 SV-H levels were found to be significantly different between benign and prostate tumor (Figure 2). AGR2 SV-C that was mainly expressed at low levels in the prostate cell lines (Figure 1B) was again less abundant in the tissue samples (Figure 2).

**Figure 3:**
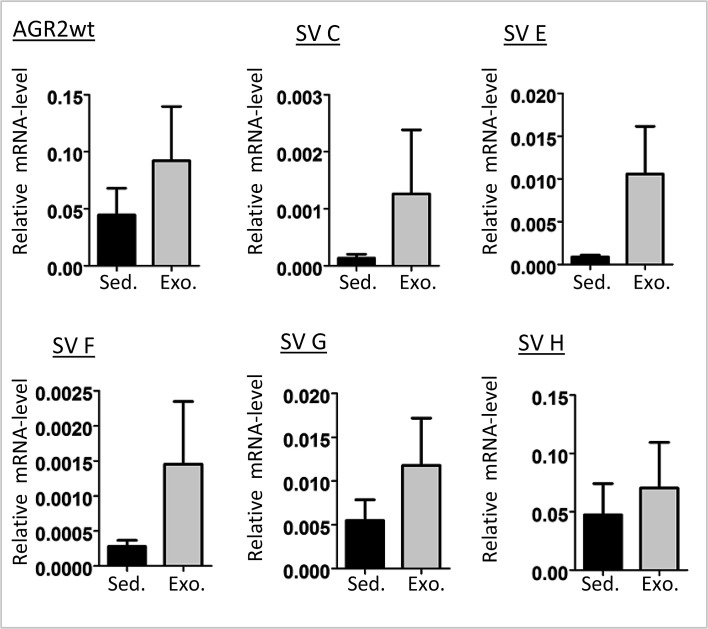
Characterization of urine exosomes Urine extracellular vesicles isolated by differential centrifugation were characterized by transmission electron microscopy and dynamic light scattering analysis. (A) Shown are the electron microscopy images of the vesicles (scale bar 500 nm) and (B and C) the size and mass distribution of the vesicles as determined by light scattering. (D) Protein lysates of urine sediments (Sed) or exosomes (Exo) from 3 prostate cancer patients used in Western blot analysis with specific antibodies against Tsg101, CD9, Hsp70, PSMA and control GAPDH specific antibody to demonstrate equal protein loading.

We next extended our analysis to extracellular membrane vesicles. These vesicles were isolated by differential centrifugation from urine of benign or PCa patients. The vesicles were negatively stained and characterized by transmission electron microscopy to be 40-100 nm diameter which corresponded to the size of exosomes [19] (Fig. 3A). Analysis by dynamic light scattering revealed a homogenous population with an average size of 74.0 ± 85.9 nm consistent with the electron microscopy data (Fig. 3B). However the mass distribution of the vesicles suggested the existence of a small proportion of larger vesicles that might have arisen from agglomeration of the single vesicles (Fig. 3C). We further analyzed the protein content of the extracellular membrane vesicles isolated from the urine of 3 prostate cancer patients and compared them to the corresponding urine sediments of the same samples. Western blot analysis identified the exosomal markers TSG101 and CD9 in the vesicle preparations but not in the sediments. The cytosolic protein Hsp70, known to be localized to exosomes of prostate cancer cells [20], was detected in both the sediment and the vesicle fractions (Figure 3C). In contrast, the endoplasmic reticulum-resident protein calnexin was detected in the sediments and not in the vesicles (Figure 3C). Based on these data, we concluded that these vesicles isolated from the urine are exosomes. Additionally, we could show that prostate-specific membrane antigen (PSMA), a prostate cancer marker [21], was enriched in the exosomal fraction (Figure 3C), indicating the prostate cancer origin of our exosome preparations.

**Figure 4:**
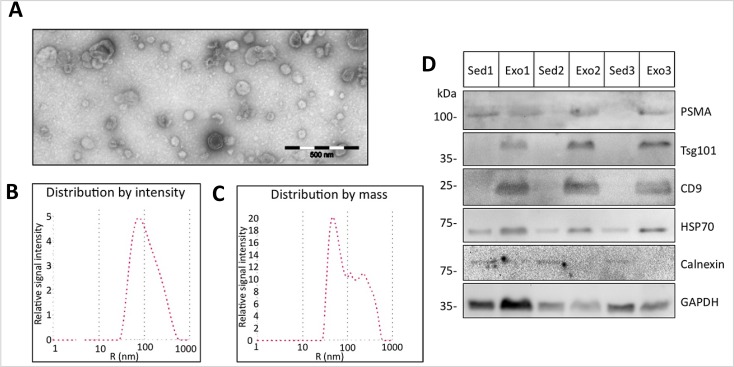
AGR2 transcript levels in urine sediments and exosomes RNA from urine sediments and exosomes from five PCa patients were used for cDNA and the expression of AGR2 wild-type and variants was analyzed. Shown as bar charts are the expression levels of the AGR2 transcripts relative to the level of expression of the human ribosomal subunit 36B4. The results are the mean ± SEM.

**Figure 5:**
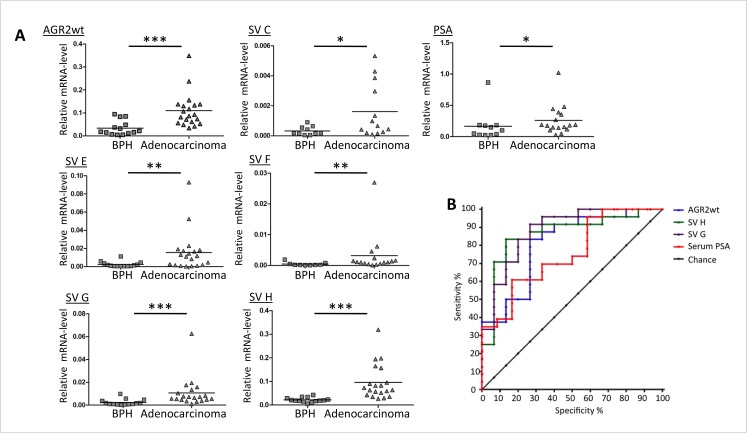
Expression of AGR2 transcripts in urine exosomes Exosomes were isolated from urine and were directly used for RNA preparation followed by qRT-PCR analysis. (A) Shown as scatter plots are the transcript levels relative to the level of expression of human ribosomal subunit 36B4. (* represents p ≤ 0.05, ** p ≤ 0.01 and *** p ≤ 0.001). (B) Receiver operator characteristic (ROC) curves of the markers urine AGR2 wt transcript, svH, svG and serum PSA.

We then performed comparative AGR2 variant transcript analyses in urine exosomes and urine sediments from 5 prostate carcinoma patients. Overall there were higher AGR2 transcript levels in the exosome fractions with no special trend towards any particular variant type (Figure 4). We therefore analyzed the expression of all the AGR2 variants in urine exosomes isolated from 24 prostate cancer patients and compared them to 15 benign samples (see Table 1 for patient characteristics). PSA transcript analysis was used in this assay as a control. In this study, all the transcripts showed a significant difference in expression in PCa compared to benign samples with AGR2 SV-H, SV-G and AGR2wt being the most significant (Figure 5A). On the basis of associations with PCa and significant pathology, ROC curves for the most significant AGR2 transcripts identified an area under the curve (AUC) scores of 0.96, 0.94 and 0.91 for AGR2 SV-H, SV-G and AGR2wt compared to serum PSA (AUC; 0.72) (Figure 5B). Comparison of the AUCs showed significance levels of 0.026 and 0.044 only for AGR2 SV-H and SV-G. Neither AGR2wt alone nor AGR2wt/PSA transcript ratio compared significantly to serum PSA levels (Figure 5B). Exosomal AGR2_SV-H and AGR2_SV-G transcript levels were therefore identified as highly significant predictive biomarkers for PCa.

**Table 1:** Descriptive characteristics of the study population

	Prostate cancer (n = 24)	Benign Hyperplasia (BPH) (n = 15)
Age [years]		
Mean / Stdev / Median	62.25 / 6.46 / 63.0	65.80 / 7.93 / 65
Range	53.0 – 71.0	54.0 – 79.0
**PSA [ng/ml]**		
Mean / Stdev / Median	11.66 / 9.08 / 7.79	5.242 / 2.847 / 5.795
Range	3.0 – 33.5	1.29 – 10.40
**Gleason Score (%)**		
6	0/24 (0.0)	-
7	21/24 (87.5)	-
8 to 10	3/24 (12.5)	-
**T-stage (%)**		
1	3/24 (12.5)	-
2	13/24 (54.2)	-
3	8/24 (33.3)	-
**N-stage (%)**		
N0	20/24 (83.3)	-
N1	4/24 (16.7)	-
**R-stage (%)**		
R0	20/24 (83.3)	-
R1	4/24 (16.7)	-
**D'amico classification (%)**		
Low	0/24 (0.0)	-
Intermediate	1/24 (4.2)	-
High	23/24 (95.8)	-

## DISCUSSION

In this work we have shown that the levels of distinct splice variants of AGR2 in urine exosomes are sensitive markers for non-invasive PCa diagnosis and outperform PSA in distinguishing between benign and PCa. We focused in this study on urine samples since urine-based diagnostic tests are non-invasive and are favorably considered over traditional needle or excision biopsies due to reduced patient pain and inconvenience. In previous studies, promising PCa biomarkers such as PCA3 and TMPRSS2:ERG have also been assessed in urine samples [22,23]. While urine PCA3 was shown to be highly specific, it has a somewhat reduced sensitivity compared to PSA [24]. In the case of TMPRSS:ERG, its major drawback as a diagnostic marker is its rather heterogeneous occurrence in cancer patients of different ethnic groups [25].

AGR2 is expressed in several human tumor tissues rich in epithelial cells, like prostate, breast, small intestine, colon, lung, and pancreas [7,26]. However reports that two splice variants of AGR2 (Δ 6 and Δ 4-6) are selectivity expressed in distinct hepatocellular neoplasms [15] provided hints that analyzing prostate cancer specific splice variants of AGR2 might improve the specificity of this biomarker. In the present study we report on the occurrence of 5 additional variants of AGR2 (AGR2 SV-C, AGR2 SV-E, AGR2 SV-F, AGR2 SV-G and AGR2 SV-H) in prostate tumor cell lines and tissues. With the exception of AGR2 SV-H, the AGR2 splice variants do not show a striking difference in expression in PCa compared to benign tissue. However in urine exosomes, some of these splice variants were shown to be selective for PCa, indicating that the abundance of mRNA in exosomes do not necessarily reflect the same pattern of expression in the tissues from which they originate. This is evident in our results from the tissue biopsies and urine exosomes. In the tissue biopsies only SV-H distinguished between PCa and benign while in the urine exosomes AGR2 SV-G and AGR2 SV-H were identified as highly specific for PCa diagnosis.

A gentle prostate massage has been reported to increase the exosomal secretion into the first-catch urinary fraction and to improve the assay for mRNA transcripts for prostate cancer diagnosis [27]. However in a recent pilot study on the level of PCA3 mRNA in urinary exosomes before and after digital rectal examination (DRE), although DRE resulted in a higher biomarker level, no diagnostic value of PCA3 could be demonstrate in the exosome fraction [28]. Our analyses also showed a lack of diagnostic power of exosomal PSA, AGR2 or even AGR2/PSA transcript ratio. However we could demonstrate a high diagnostic power of exosomal AGR2 SV-G and SV-H even in the absence of any manipulations of the prostate.

One problem in all clinical studies in prostate cancer diagnosis is the definition of tumor free control groups. As we compared AGR2 splice variant to PSA levels in our study, a control group derived from patients with low PSA levels could not be used. Instead we used a control group based on negative histology results obtained either from negative prostate biopsy (performed due to elevated PSA values) or after transurethral resection (if PSA values were low and prostate biopsy was considered inappropriate according to the guidelines). Ethical limitations did not allow biopsies to be taken from healthy patients in the setting of this pilot study. We therefore used as benign control, samples from tumor free patients who underwent transurethral resections performed as a wide resection, preserving just the prostate capsule and small parts of the peripheral zone. However in further clinical studies all patients (including the control group) should be biopsied. Despite these apparent limitations and the relatively small sample size of this study, our findings on the diagnostic power of urinary exosomal AGR2 SV-G and SV-H levels strongly encourage future clinical studies to further validate the significance of these potentially new biomarkers.

## METHODS AND MATERIALS

### Cell culture

Human cancer cell lines LNCaP, 22Rv.1, PNT2, HEK293T and HeLa were obtained from ATCC. MCF7 and HepG2 cells were provided by Gunther Schütz (Heidelberg, Germany) and Gary Ryffel (Essen, Germany) respectively while Ishikawa and JEG-3 cells were provided by Miguel Beato, Marburg, Germany. All cells were cultured in Dulbecco modified Eagle's medium supplemented with 10% fetal calf serum (FCS) at 37°C and 5% CO_2_ with the exception of LNCaP and PNT2 cells which were cultured in RPMI 1640 medium supplemented with 10% FCS. All cell lines used in this study were last authenticated by short tandem repeat analysis on December 16, 2013.

### Clinical samples

This study was approved by the local ethics committees of the Innsbruck Medical University, Austria and the Medical Center-University of Freiburg, Germany and performed in accordance with the ethical standards laid down in the 1964 Declaration of Helsinki and its later amendments. Frozen prostate tissue samples from previously untreated patients who had undergone radical prostatectomy after tumor diagnosis in a PSA-based prostate cancer early detection program were obtained from the prostate center of the Department of Urology of the Innsbruck Medical University [29]. Frozen tissue tumor samples were obtained from a cohort of Gleason score (GSC) 6 tumors (Gleason pattern 3) and a group of GSC 8–10 tumors (Gleason patterns 4 and 5). The analysis of urine exosomes was performed with samples from 39 patients: 24 with prostate cancer (as proven by a positive prostate biopsy) and 15 with benign prostate hyperplasia. The control group included individuals presenting for urological check-up or treatment of prostate hyperplasia. The patients of the control group had negative digital rectal examination and either negative prostate biopsy or benign histology results after transurethral resection. Urine samples were provided by the Department of Urology, University Medical Center Freiburg. First void urine was collected prior to any intervention and stored immediately at 4°C before processing, which was done within 12 h.

### Isolation and characterization of of urine exosomes

Urine sediments were obtained from 50 ml urine by centrifugation at 2500 x g (30 min, 4°C), washed with phosphate buffered saline (PBS) and re-collected by centrifugation at 10,000 x g (5 min, 4°C). To harvest exosomes, the supernatant from the first centrifugation step was diluted 1:1 with PBS, centrifuged at 10,000 x g (30 min, 4°C) and filtered through a 0.2 μm filter unit to remove residual cell debris and larger vesicles. The exosomes were collected by ultracentrifugation at 120,000 x g (90 min, 4°C) using SW41 rotor (Optima LE-80K Ultracentrifuge, Beckman Coulter, Krefeld, Germany), resuspended either in PBS (for protein analysis and exosomes characterization) or directly in RLT buffer (Qiagen, Hamburg, Germany) for RNA isolation. The urine exosomes were analyzed by dynamic light scattering (DLS) using a Zetasizer Nano ZS (Malvern Instruments, Herrenberg, Germany) according to the manufacturer's instructions. The quality of exosome preparation was ascertained by electron microscopy. The samples were loaded on a 300-mesh copper grids, fixed with 1% glutaraldehyde, washed with doubled distilled water and negatively stained with one drop of 1% uranyl acetate.

### RNA isolation, and cloning of AGR2-splice variants

RNA from cultured cells was isolated using PeqGold RNApure (Peqlab, Erlangen, Germany) while RNA from prostate tissues was isolated and amplified as previously described [30]. RNA from urine sediments and exosomes was isolated using RNeasy Mini Kit (Qiagen). Cellular RNA was reverse transcribed into cDNA with random primers using the M-MLV reverse transcriptase (Promega Mannheim, Germany). Exosome RNA was reverse transcribed using SuperScript® VILO™ cDNA Synthesis Kit (Life Technologies Darmstadt, Germany). AGR2 splice variants were amplified using the following primers: AGR2 exon1L fw: 5'- GGG GAA AGG AAG GTT CGT TTC -3' (AGR2 SV-C); AGR2 exon1 fw: 5'- CGA CTC ACA CAA GGC AGG T -3' (AGR2 SV-E, -F, -G); AGR2 exon 2 fw: 5'-ATG GAG AAA ATT CCA GTG TCA GCA-3' (AGR2 SV-H); AGR2 exon 8 rev: 5'-TCC ACA CTA GCC AGT CTT CTC A-3' (AGR2 SV-C, -E, -F, -G, -H ).

Sequences were cloned using the pCR2.1 Topo cloning kit (Life Technologies Darmstadt, Germany) and sequenced by Sanger dideoxy sequencing (Genebank accsession numbers: AGR2_SV-C: KJ767789; AGR2_SV-E: KJ767791; AGR2_SV-F: KJ767792; AGR2_SV-H: KJ767793).

### Quantitative real time PCR

Quantitative real-time PCR was carried out using the GoTaq SyBr green qPCR-mix (Promega, Mannheim, Germany). Forward/backward primer pairs used are: PSA fw: 5'-ACCAGAGGAGTTCTTGACCCCAAA- 3'; PSA rev: 5'-CCCCAGAATCACCCGAGCAG-3'; Rib36B4 fw: 5'-GAA GGC TGT GGT GCT GAT GG-3'; Rib36B4 rev: 5'-CCG GAT ATG AGG CAG CAG-3'; SV-A fw: 5'-GCCAACAGACAACCCAAAGT-3'; SV-A rev: 5'-GCAAGAATGCTGACACTGGA-3'; SV-B (AGR2wt) fw: 5'-CGACTCACACAAGGCAGGT-3'; SV-B (AGR2wt) rev: 5'-GCAAGAATGCTGACACTGGA-3'; SV-C fw: 5'-CAC AAG GCA GAG TTG CCA TGG-3'; SV-E fw: 5'-ATC TGG TCA CCC ATC TCT GA-3'; SV-F fw: 5'-GGA AAT CCA GAC CCA TCT CTG-3'; SV-G fw: 5'-AAG GCA GGT ACA GCT CTG-3'; Reverse SV-C, -E, -F -G: 5'-TCC ACA CTA GCC AGT CTT CTC A-3'; SV-H fw: 5'-ATG GAG AAA ATT CCA GTG TCA GCA-3'; SV-H rev: 5'-ACT TGA GAG GTT CTT CAT ATG TCT G-3'(Note that SV-H rev used in this study carries a sequence alteration that was identified in the first clone sequenced for SV-H. However as other sequenced clones lack this alteration, this would suggest the use of 5'-ACT TGA GAG CTT TCT TCA TAT GTC TG-3'as SV-H rev primer in future studies. This would be in accordance with nucleotides 378-353 of the sequence submitted to Genebank (KJ767793)).

### Western Blotting

Western blotting was carried out using the following antibodies: Calnexin (New England Biolabs, Frankfurt am Main, Germany); HSP-70, tsg-101, CD9 and GAPDH (Santa Cruz Biotechnology, Heidelberg, Germany); anti-PSMA mAb 7E11-C5 was purified from a hybridoma supernatants (ATCC, Manassas, VA, USA) as previously described [31].

### Statistical evaluation

Differences among AGR2 expression levels in tissue and in urine sediments were analyzed using the Mann–Whitney U-test (non-normally distributed data). Student's t-test was used to test differences in all other experiments. Logistic regression was performed for each variable or ratio of variables. The calculated probabilities were plotted as receiver operating characteristic (ROC) curves to display sensitivity and specificity of urine AGR2 transcript variables and ratios. Area under the curve (AUC), standard error (SE), 95% confidence interval (95%CI) and p-value were calculated for each ROC curve to determine statistically significant differences. We used MedCalc Version 12.7.8.0 for statistical analysis.
